# Comparison of artificial intelligence methods with manual measurements for mandibular bucco-lingual bone width assessment using cone-beam computed tomography images

**DOI:** 10.1007/s11282-026-00916-8

**Published:** 2026-03-24

**Authors:** Hanna N, Junaid Ahmed, Nanditha Sujir, Tushar Verma, Kathan Vakharia, Jeny Rajan, Nandita Shenoy

**Affiliations:** 1 Department of Oral Medicine and Radiology, Manipal College of Dental Sciences Mangalore, Manipal Academy of Higher Education, Manipal, India; 2 Department of Computer Science and Engineering, National Institute of Technology Karnataka, Surathkal, Mangalore, Karnataka 575025 India

**Keywords:** Artificial intelligence, Bone density, Cone-beam computed tomography, Deep learning, Mandibular canal, Neural networks

## Abstract

**Objectives:**

Accurate bucco-lingual bone width measurement is essential for implant planning. Manual cone-beam computed tomography (CBCT) assessment is time-intensive and operator-dependent. This study aimed to evaluate the accuracy of an artificial intelligence (AI)–based system for automated bone width measurement compared with manual methods.

**Methods:**

A retrospective diagnostic accuracy study was conducted using 300 CBCT scans of posterior mandibular edentulous sites. Manual bucco-lingual bone width was measured at 2-mm intervals from the alveolar crest to 2 mm superior to the mandibular canal. A deep learning framework with U-Net + + was trained to segment the alveolar ridge and mandibular canal, followed by automated bone width measurements. Model performance was assessed using Dice score, Intersection over Union (IoU), precision, and recall for segmentation accuracy, and regression metrics (mean squared error [MSE], mean absolute error [MAE], root mean squared error [RMSE], and coefficient of determination [R²]) for comparison with manual measurements.

**Results:**

U-Net + + demonstrated high accuracy for alveolar ridge segmentation (Dice score, 0.9798; IoU, 0.9606; precision, 0.9820; recall, 0.9778) and moderate accuracy for mandibular canal segmentation (Dice score, 0.5640). Automated bucco-lingual width measurements showed reasonable correspondence with manual values (MSE, 1.9700 mm²; MAE, 1.1900 mm; RMSE, 1.4000 mm; R², 0.5300). Qualitative analysis confirmed high visual correspondence for ridge segmentation, though variability persisted in canal delineation.

**Conclusions:**

The AI-based U-Net + + system reliably segmented the alveolar ridge and provided bone width measurements with moderate agreement to manual methods. Mandibular canal segmentation remained a limitation. Broader validation across jaw regions and imaging systems is recommended to enhance clinical utility.

## Introduction

Accurate evaluation of alveolar bone morphology is critical for successful dental implant placement. Traditional two-dimensional radiographs are limited in depicting bone patterns due to the overlapping of three-dimensional structures in images. Cone-beam computed tomography (CBCT) has become the standard in oral and maxillofacial imaging due to shorter, three-dimensional imaging exposure times, lower radiation dose, and cost-effectiveness [1]. CBCT provides high-resolution submillimetre images, with precise information on vital structures, implant site dimensions, bone density, and ridge profiles, thereby reducing implant failure rates [2, 3].

Despite these advantages, CBCT interpretation for implant placement can be cumbersome due to the requirement of time-intensive manual measurements. Analysis requires specialist training due to the large image volume. Artificial intelligence (AI) offers potential to reduce interpretation time by rapidly identifying implant sites and extracting measurements. Deep learning, particularly convolutional neural networks (CNNs), has enhanced diagnostic efficiency and reliability [4].

Several studies in the literature have examined the applications of AI in implant planning. Al-Sarem et al. [5] developed a CNN with 500 CBCT scans for missing tooth detection. In this study, DenseNet169 achieved the highest precision (0.98) with segmentation accuracy of 93.3% and classification accuracy of 89%. Alsomali et al. [6] looked at the identification of stent GP markers for implant planning and found an 83% true-positive rate, 2.8% false-positive rate and 17% missed GP markers. Hedayatipanah et al. [7] reported that metal artefact reduction algorithms improved peri-implant bone width accuracy, with Cranex 3D outperforming ProMax 3D. Bayrakdar et al. [3] found that the Diagnocat AI system [8] showed no statistical difference in comparison to manual linear measurement. However, it showed a significant difference in measurement of alveolar width. Missing teeth were detected with 95.3% accuracy, canals with 72.2% and sinuses with 66.4%. However, it was noted that high costs may limit clinical adoption. Bodhe et al. [9] designed an AI framework to predict implant success and optimise implant design via finite element analysis, underscoring AI’s role in personalised planning.

CNN-based segmentation architectures such as U-Net [10], U-Net++ [11], Double U-Net [12], and DeepLab [13] are commonly used for medical image segmentation and have advanced automated analysis of dental CBCT images by enabling precise delineation of anatomical structures and abnormalities through encoder–decoder designs, skip connections, and adversarial or multi-scale feature learning strategies.

Since accurate bone width estimation is vital for implant stability and avoidance of complications such as cortical plate perforation, validation of AI-derived measurements against manual methods is clinically relevant.

Although artificial intelligence has been increasingly incorporated into implant planning workflows, most published studies have concentrated on detection, classification, and segmentation tasks, including missing tooth identification, implant site localisation, and anatomical structure segmentation. In contrast, comparatively little attention has been given to the direct quantitative validation of automated measurements derived from CBCT data. In particular, the automated assessment of bucco-lingual alveolar bone width has not been systematically evaluated as a continuous measurement task against manual CBCT measurements using clinically defined anatomical reference points.

The present study addresses this gap by developing a fully automated deep learning–based framework that combines U-Net++–based segmentation of the alveolar ridge and mandibular canal with anatomically constrained bucco-lingual bone width computation at fixed vertical intervals referenced to clinically relevant landmarks. Rather than limiting evaluation to segmentation performance alone, this study directly examines the correspondence between AI-derived and manually obtained CBCT measurements using regression-based statistical analysis.

By focusing on quantitative measurement performance rather than structure detection alone, this work aims to move AI applications in implant planning toward clinically meaningful measurement support and decision-making integration.

### Aim

To assess the accuracy of AI-based bucco-lingual bone width measurements for implant planning and compare them with manual measurements across small, medium, and large field-of-view (FOV) CBCT images.

## Materials and methods

This study was approved by the institutional ethics committee (protocol No. 23036; clinical trial number: not applicable) and conducted as a retrospective diagnostic accuracy study in accordance with institutional guidelines. CBCT scans were anonymised prior to analysis. Owing to the retrospective design and de-identified data, informed consent was waived.

### Inclusion criteria

- CBCT images of posterior mandibular edentulous sites, including both single-tooth and multiple-tooth edentulous regions.
- Small, medium, and large field-of-view (FOV) scans, reconstructed with standardized voxel sizes (0.2–0.3 mm) and uniform reconstruction parameters.
- High-resolution, clear cross-sectional images suitable for segmentation and measurement.

### Exclusion criteria

- Non-edentulous or fully dentate mandibular regions.
- Images with artefacts, poor resolution, or inadequate anatomical clarity.
- Incomplete or misaligned CBCT scans excluding bucco-lingual measurement sites.
- Pathological sites affecting measurement accuracy or segmentation.

CBCT scans were obtained for implant planning using a Planmeca ProMax 3D CBCT unit (Planmeca Oy, Helsinki, Finland). The scans included small, medium, and large fields of view (FOVs), selected according to the clinical indication. In the present study, small FOV corresponded approximately to volumes of 40 × 50 mm to 50 × 50 mm, medium FOV to approximately 80 × 80 mm to 100 × 100 mm, and large FOV to volumes ≥ 160 × 100 mm, consistent with the acquisition presets available in the Planmeca ProMax 3D system.

Exposure parameters varied within the manufacturer’s preset range (approximately 84–90 kV, 6–10 mA, and 12–14 s exposure time) depending on the chosen FOV and patient size. Images were reconstructed with a voxel size of 0.2–0.3 mm and analyzed in cross-sectional slices perpendicular to the alveolar ridge using Romexis software version 4.6.2.

To minimise variability related to FOV differences, all scans were reconstructed using standardized voxel sizes, uniform reconstruction algorithms, and consistent cross-sectional orientation perpendicular to the alveolar ridge, ensuring comparable spatial resolution and measurement geometry across datasets.

Cross-sectional slices were generated using standardized reconstruction protocols, with slice orientation aligned perpendicular to the long axis of the alveolar ridge and referenced to consistent anatomical landmarks. Slice selection was based on ridge morphology at posterior mandibular edentulous sites, ensuring uniform angulation and spatial orientation across cases. For each eligible edentulous site, cross-sectional images were extracted at standardized positions along the ridge, with consistent slice thickness and spacing to minimise geometric variation.

### Manual measurements

Cross-sectional slices were evaluated by a radiologist (H.N.). The number of cross-sectional slices per CBCT corresponded to the number of posterior mandibular edentulous sites meeting inclusion criteria. For each site, measurements were obtained from standardized cross-sectional views positioned through the center of the edentulous ridge span, ensuring consistent anatomical location and measurement geometry. Bucco-lingual width was defined as the distance between the external buccal and lingual cortical plates, measured perpendicular to the ridge axis. Measurements were recorded at the alveolar crest and at 2-mm intervals apically until 2 mm superior to the mandibular canal. Each site was measured twice at separate time points by a single trained radiologist (H.N.), and the mean values were recorded. To ensure intra-operator reliability and measurement reproducibility, standardized anatomical landmarks, fixed measurement definitions, and consistent slice orientation criteria were applied across all cases. The use of uniform measurement protocols and repeated measurements minimized operator-dependent variability. All measurements were performed perpendicular to the ridge axis to maintain geometric consistency and reduce variability related to ridge morphology and edentulous span length.

### Sample

A total of 300 CBCT scans meeting the inclusion criteria were retrospectively retrieved and included for analysis. Axial sections of posterior mandibular edentulous sites were extracted from all scans. The entire dataset (*n* = 300) was utilized for analysis, with 80% allocated for model training and 20% for validation. The sample size was determined in consultation with a statistician based on feasibility and comparability with similar AI imaging studies.

The dataset included both single-tooth and multiple-tooth posterior mandibular edentulous sites. To control for morphological variability associated with differing resorption patterns, all measurements were performed using standardized anatomical reference points (alveolar crest and mandibular canal) and fixed vertical intervals (2-mm increments), ensuring consistent measurement geometry across all edentulous configurations.

### Rationale for posterior mandible

This region was selected to maintain dataset consistency and because anatomical features such as lingual concavities and canal proximity render precise bucco-lingual measurements clinically significant.

### AI framework

**Fig. 1:**
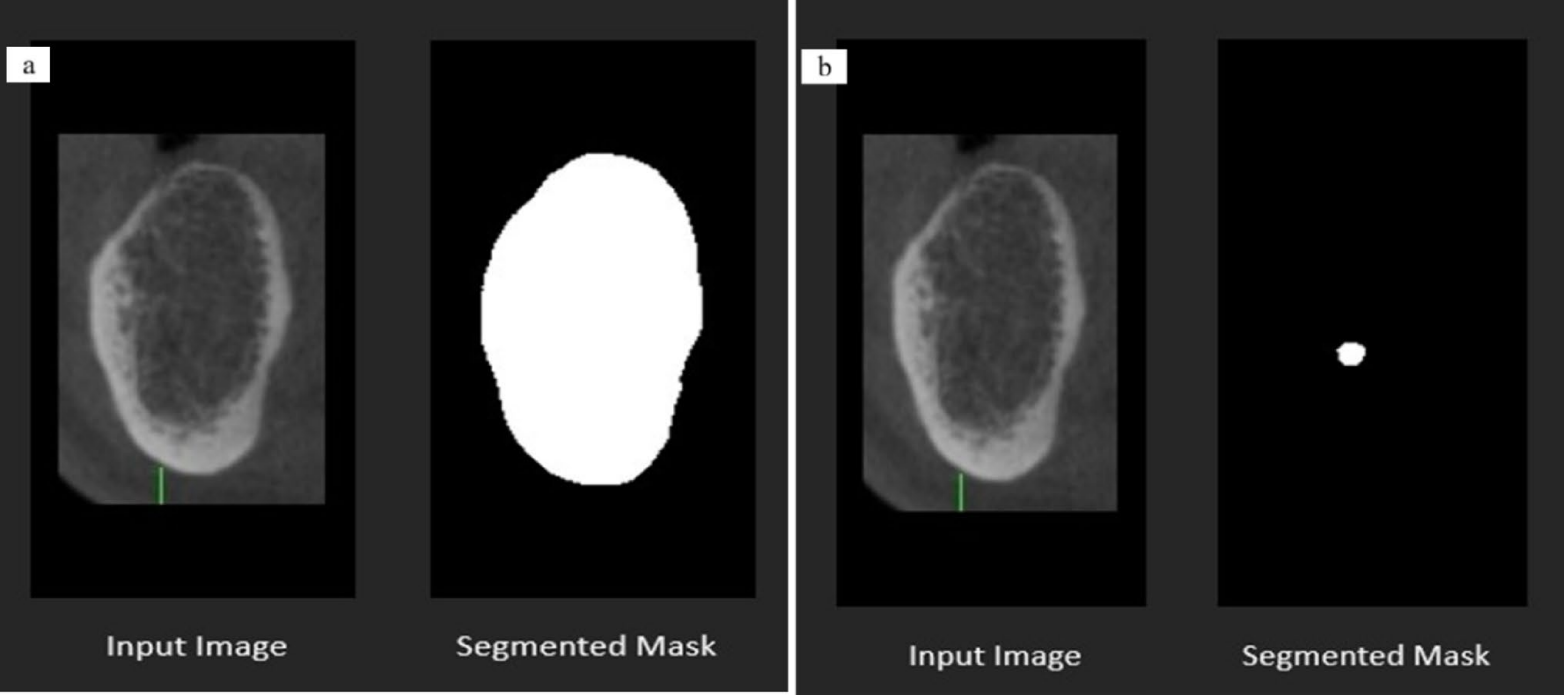
Illustrations of the input images and segmented regions. **a** area of interest (AOI) segmented region; **b** nerve segmented region

**Fig. 2:**
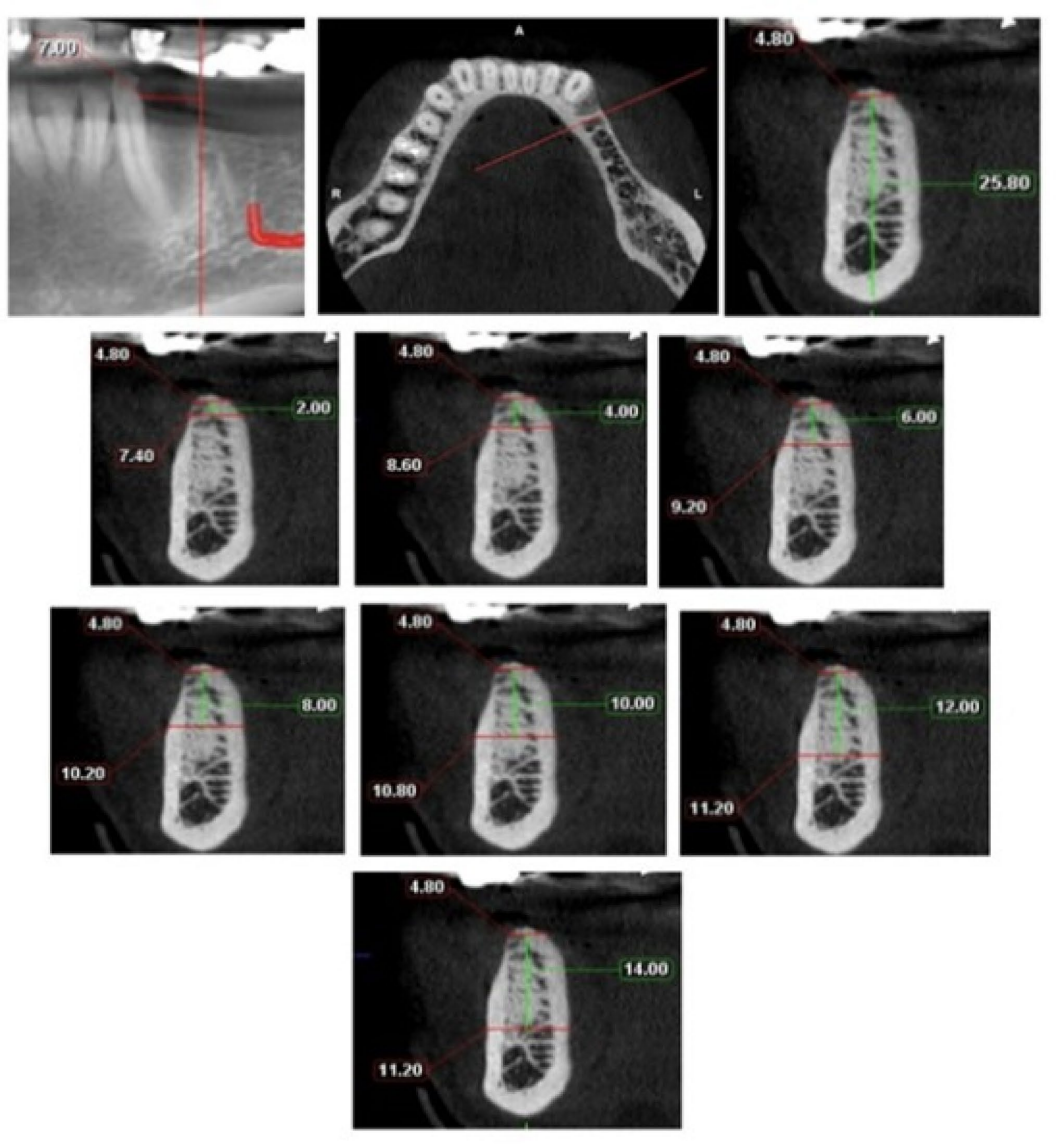
Demonstration of width calculation in the segmented canal region. Annotated measurements are shown at regular intervals to provide precise and interpretable data

Data were blinded prior to processing. Multiple CNN architectures (U-Net, Double U-Net, U-Net++, DeepLab) were tested, with U-Net + + selected for segmentation tasks. We selected the U-Net + + architecture after conducting an ablation study with all the aforementioned architectures. All these models were trained from the scratch. The models differ primarily in how they learn and combine features at different levels of detail. DeepLab V3 + focuses on capturing the overall shape of large regions but may miss very thin structures. The Double U-Net adds a second refinement stage to improve detail, although it becomes more complex and sensitive to noise. U-Net with EfficientNet improves feature learning but still has limited detail merging. U-Net + + utilizes more connected pathways to gradually refine features, resulting in clearer boundaries and more accurate overall results. In this experiment, U-Net + + with a depth of 5 was used for both alveolar ridge (AOI) and mandibular canal segmentation. The architecture consisted of four encoder levels, a bottleneck layer, and the corresponding decoder levels. Data augmentation included horizontal and vertical flips, shift–scale–rotate transformations, elastic deformation, and random brightness adjustment. Model training was performed using the Adam optimizer with a learning rate of 0.001 and binary cross-entropy as the loss function. The model was trained for 100 epochs, and the best-performing checkpoint was retained for inference. The proposed model segmented the alveolar ridge and inferior alveolar nerve. Ground truth segmentation masks were generated using a semi-automated annotation pipeline to reduce subjective variability. CBCT cross-sectional images containing color-coded anatomical annotations were converted to HSV color space to enable robust threshold-based segmentation. Contour detection was subsequently applied, and the largest connected component was selected as the anatomical region of interest. Minor manual refinements were performed only when image artefacts disrupted contour continuity. This approach ensured annotation consistency across the dataset while minimizing manual bias. Automated bucco-lingual width was then measured at 2-mm intervals from the alveolar crest to the canal. All bucco-lingual width measurements were computed fully automatically from predicted segmentation masks. Measurements were extracted at fixed 2-mm intervals using geometric distance calculations between buccal and lingual cortical boundaries. No post-segmentation manual correction or operator interaction was performed, ensuring reproducibility and eliminating user-dependent variability. Figures 1a, b and 2 illustrate the segmentation and measurement process.

AI-generated measurements were compared with manual measurements to evaluate accuracy. Statistical analyses were conducted to assess agreement between the methods and to determine the significance of observed differences.

### Statistical analysis

AI-generated measurements were compared with manual measurements to evaluate accuracy and correspondence between the two methods.

Quantitative segmentation performance was evaluated using Dice score, IoU, precision, and recall. Dice and IoU describe how well the segmented regions produced by the model overlap with the expert-annotated regions. Precision reflects how accurately the model identifies the area of interest without including extra regions, while recall indicates how completely the model captures the true region without missing parts. Together, these measures provide an overall understanding of both the correctness and completeness of the segmentation and offer a comprehensive assessment of the model’s ability to delineate the area of interest (AOI) and the mandibular nerve regions.

To assess the model’s accuracy in bone width measurement, we used regression-based metrics including MSE (mm²), MAE (mm), RMSE (mm), and R². These metrics compare the model’s predicted measurements with manual clinical measurements obtained at 2 mm vertical intervals from the alveolar crest toward the mandibular canal. Lower values of MSE, MAE, and RMSE indicate smaller measurement errors and quantify the magnitude of error in clinically interpretable units, while a higher R² value reflects stronger agreement between the AI predictions and the manual reference measurements. R² was additionally used to assess the linear correlation between the two methods.

A paired t-test was also performed to compare AI-derived bucco-lingual width measurements with the corresponding manual measurements in order to assess whether a statistically significant difference existed between the two methods. However, more detailed agreement analyses, such as Bland–Altman evaluation, were not performed and are identified as an important direction for future validation in larger, prospectively designed datasets.

All evaluations were conducted on the independent test dataset. Descriptive statistics were reported to summarise performance across test cases. Given the exploratory and pilot nature of this study, primary emphasis was placed on quantitative error metrics to characterize measurement performance. All computations were performed using Python-based libraries, including NumPy, SciPy, and scikit-learn.

## Results

1. *Segmentation performance*.
  The U-Net + + model was quantitatively evaluated on the test dataset for both AOI and mandibular nerve segmentation. Among the evaluated architectures (U-Net, Double U-Net, DeepLabV3+, and U-Net++), U-Net + + demonstrated the highest overall segmentation performance and was therefore selected for subsequent analysis.
  Dice score: 0.9798 for AOI and 0.5640 for the nerve region.
  IoU score: 0.9606 for AOI and 0.4194 for the nerve region.
  Precision: 0.9820 for AOI and 0.5880 for the nerve region.
  Recall: 0.9778 for AOI and 0.5819 for the nerve region.
  These findings indicate high accuracy in AOI segmentation, with moderate performance in mandibular nerve segmentation, likely due to anatomical complexity and low contrast in the canal region (Table 1). Mandibular nerve segmentation was primarily used to identify the reference location for initiating the bucco-lingual width calculations. On the other hand, the results shown discuss the proper match with the predicted output and ground truth, hence finding the most mapped region is more important than the numerical values. The loss curves of both models (alveolar ridge segmentation and mandibular canal segmentation) are shown in Fig. 5.
  **Table 1:** Quantitative segmentation performance of the final selected model (U-Net++) for AOI and mandibular nerve segmentation

Metric	AOI	Nerve
Dice Score	0.9798	0.5640
IoU Score	0.9606	0.4194
Precision	0.9820	0.5880
Recall	0.9778	0.5819
2. *Measurement accuracy*.
  AI-based measurements of bucco-lingual bone width were compared with manual measurements. Regression analysis produced the following metrics:
  Mean squared error (MSE), 1.9700 mm².
  Mean absolute error (MAE), 1.1900 mm.
  Root mean squared error (RMSE), 1.400 mm.
  Coefficient of determination (R²), 0.5300.
  These findings demonstrate acceptable measurement accuracy, with moderate correspondence between automated and manual readings (Table 2). The output of width calculation at various heights from AOI segmentation is shown in Fig. 3. A paired statistical comparison was also performed between AI-based and manual measurements on the test dataset. A paired t-test showed: Mean difference (AI − Manual): 0.75 mm, Standard deviation of differences: 1.34 mm, t-statistic: 3.47 and p-value: 0.0013. Although the difference is statistically significant, the absolute error magnitude remains small (~ 1.19 mm MAE) and within clinically acceptable limits for bucco-lingual width assessment.

**Table 2:** Regression metrics based on 19 test images

Metric	Value
Mean Squared Error (MSE, mm²)	1.9700
Mean Absolute Error (MAE, mm)	1.1900
Root Mean Squared Error (RMSE, mm)	1.4000
R-Squared (R²)	0.5300

**Fig. 3:**
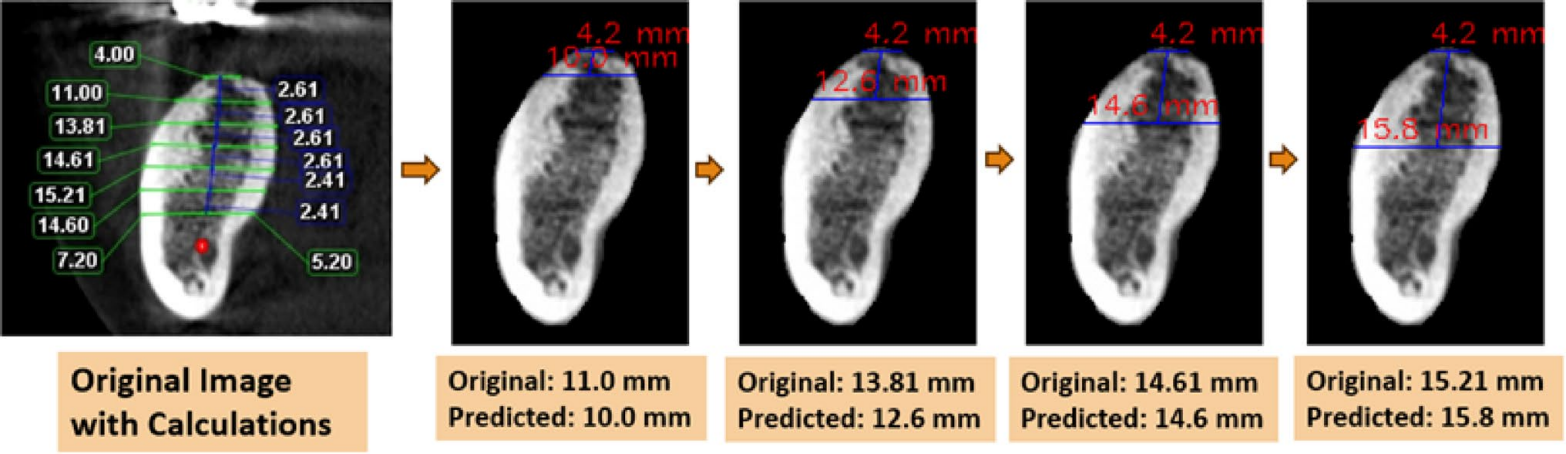
Output of width calculation at various heights from AOI segmentation

### Qualitative analysis

**Fig. 4:**
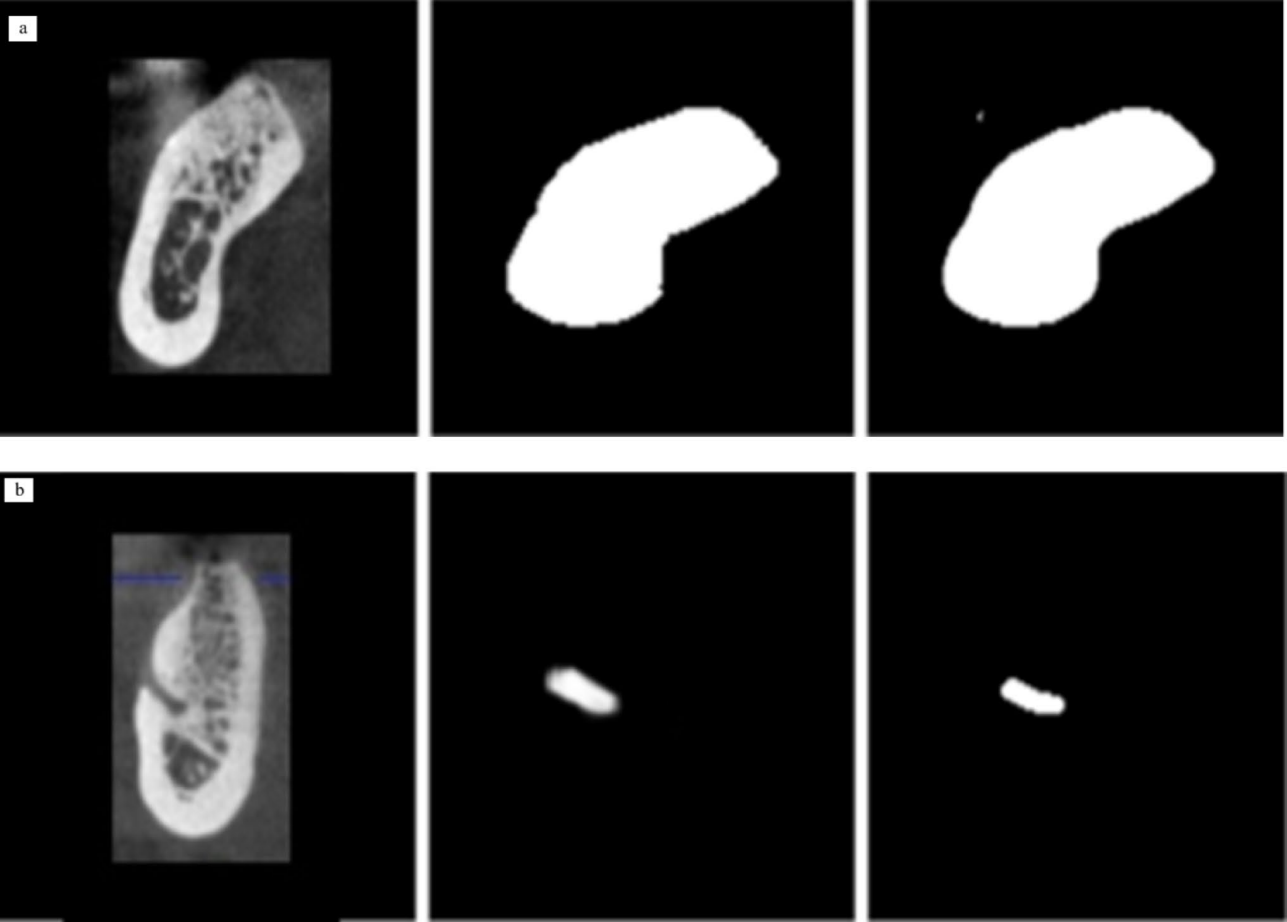
Qualitative analysis of segmentation results. Each column shows (left to right) the input image, ground truth mask, and predicted mask. **a** area of interest (AOI) segmentation; **b** nerve segmentation

**Fig. 5:**
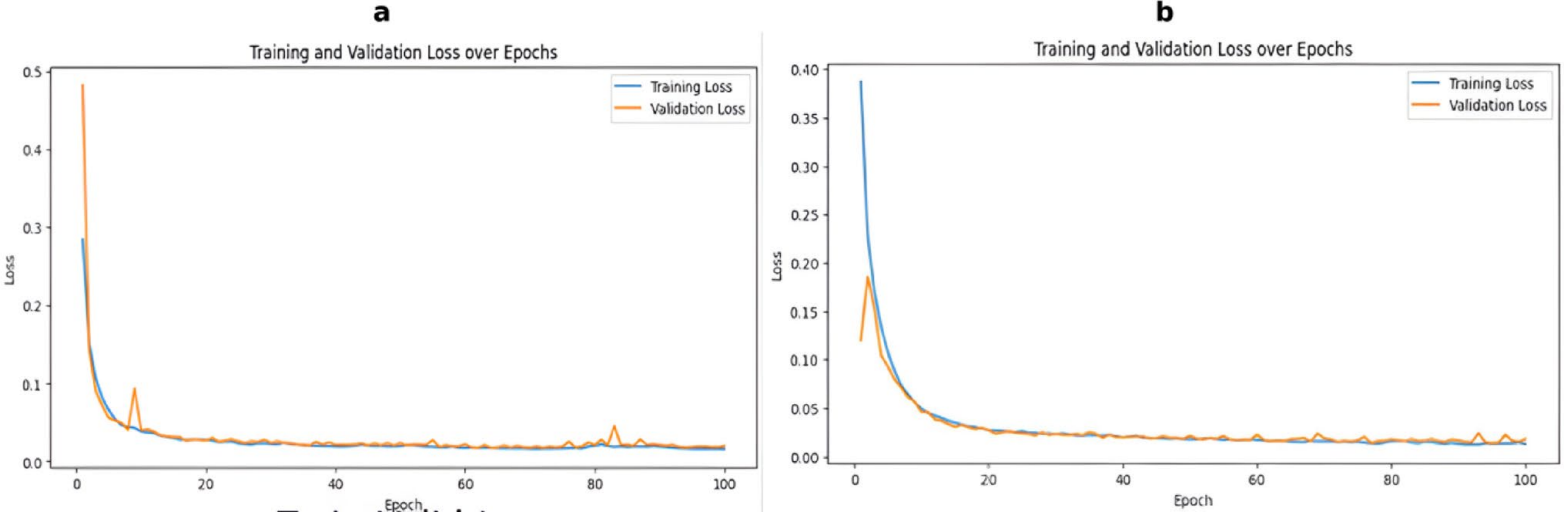
Training and Validation curve of the models for **a** alveolar ridge segmentation; **b** mandibular canal segmentation

Qualitative analysis was performed by visually comparing the predicted segmentation masks with the ground truth annotations to assess boundary accuracy. Sample visual comparisons between predicted segmentation masks and ground truth annotations demonstrated high concordance for the AOI, with greater variability observed in nerve region predictions (Figure 4a and b). The results are derived using the mapping techniques, which demonstrate the percentage of overlapped regions based on the predicted values and ground truth. The visual difference between the segmentation masks and the original CBCT images reflects the binary representation of annotated regions and the inherently low contrast of the mandibular canal, rather than annotation inconsistency (see Fig. 5).

These findings indicate that the proposed U-Net + + model achieved high accuracy in segmenting clinically relevant regions, particularly the AOI, with consistent performance across evaluation metrics. Nerve-overlapped areas helped identify the mapped areas in the ground truth region, which in turn aided in determining the region where the nerve would be available. Scores depend on the percentage of overlapping regions, whereas the actual use case depends on identifying the nerve region, which helped us make the calculations and achieve the final results.

## Discussion

The use of AI in CBCT-based implant planning is becoming increasingly relevant in modern dental practice. It offers a way to improve the accuracy of measurements, segmentation, and overall diagnostic efficiency. In this study, the U-Net + + model was able to segment the region of interest effectively and provide automated bone width measurements that were in good agreement with manual assessments. This shows the potential of such systems to assist clinicians by reducing the time and variability associated with manual interpretation [14].

Accurate assessment of bone width is essential for selecting the correct implant dimensions and avoiding complications such as cortical plate perforation or nerve injury [15]. While 2D imaging has certain limitations in terms of resolution and depth perception, CBCT provides the required three-dimensional detail for these evaluations. However, manual analysis of CBCT data can be time-consuming. AI-based systems can help by automating the process and providing consistent results, which in turn supports clinical decision-making and workflow efficiency [16].

The relatively lower segmentation performance for the mandibular canal reflects a common issue in dental imaging. The inferior alveolar canal is often difficult to identify due to variations in anatomy and low contrast between surrounding structures [17, 18]. Future improvements such as the use of transfer learning or combining multiple imaging modalities could help enhance segmentation accuracy in this area [19].

The choice of U-Net + + was based on its architectural advantages over the conventional U-Net model. Its nested skip connections improve feature propagation and help the network learn fine anatomical details [20]. Previous studies have shown that U-Net + + performs well in medical image segmentation, including head and neck regions [21]. The ability to achieve pixel-level segmentation allows bone-width measurements at specific intervals, which is particularly valuable for implant design and surgical guide fabrication. Other studies using U-Net models for alveolar bone segmentation have also reported high accuracy and minimal volumetric error, which is consistent with the present findings [22].

Using manually verified training data and scans with different fields of view strengthened the reliability of the dataset and the generalizability of the model. However, manual annotation is still a major challenge and limits large-scale dataset preparation [23]. In the future, approaches such as synthetic data augmentation and semi-supervised learning could help overcome this limitation by reducing the dependence on manual input [24].

From a clinical point of view, AI-based CBCT analysis can save considerable time and make implant planning more consistent, especially for less-experienced clinicians [25]. Although there was variability in nerve segmentation, the overall accuracy of AI-assisted bone width measurements suggests that such systems could be valuable as supportive tools in clinical practice. Further validation with larger and more diverse datasets is recommended to confirm the generalizability of these findings.

### Limitations

This study has several limitations. This study focused only on posterior mandibular edentulous sites and used CBCT scans from a single Planmeca ProMax 3D system, which may limit generalizability to anterior mandibular or maxillary regions and to scans from other imaging devices. To enhance external validity, future studies should include anterior and maxillary regions as well as datasets from multiple CBCT systems, allowing assessment of cross-device performance and broader applicability of the AI model.

Soft-tissue artefacts and patient movement may still compromise CBCT quality, potentially affecting AI performance [26].

Although U-Net + + achieved high alveolar ridge segmentation accuracy, mandibular canal detection was moderate, which could be due to the anatomical variability of the structure. Additionally, the canal’s small diameter, low contrast against surrounding bone, and anatomical variation between patients make it difficult for the model to consistently detect it. As a result, subtle canal edges may be under segmented or missed, indicating that additional strategies, such as attention-based feature enhancement or anatomical guidance, may be needed to further improve canal segmentation. In the present framework, mandibular canal segmentation was used only to identify the inferior measurement boundary rather than for precise nerve delineation; therefore, partial localization was sufficient for the intended automated bone width measurement task.

Manual measurements performed by a single trained radiologist were used as the ground truth, minimizing inter-observer variability while potentially introducing observer-dependent bias and limiting reproducibility.

Although a paired t-test was performed to assess statistical differences between AI-derived and manual measurements, formal agreement analyses such as Bland–Altman evaluation were not conducted, which limits definitive conclusions regarding measurement interchangeability between the two methods.

Other clinical parameters, including bone density, bone quality, and pathological changes, were not included in this study. Consequently, the model’s current scope is limited to bucco-lingual bone width assessment, and its comprehensive clinical applicability for implant planning is restricted. Future work could integrate these additional parameters to enhance clinical utility and generalizability.

Ethical considerations and patient data privacy must be addressed when deploying AI in clinical environments. Model transparency, explainability, and regulatory compliance are essential. Recent regulatory frameworks, including the EU AI Act and US FDA lifecycle guidance, provide evolving guidance for validation and deployment of AI-based diagnostic tools [27–29].

### Future directions

Further work will expand model training and testing across multi-arch, multi-centre datasets to ensure anatomical and technical diversity. Inclusion of CBCT data from different machines and populations would enhance external validity. Additionally, in the future, the integration of multimodal data sources—such as intraoral scans, panoramic radiographs, or digital models—could support more comprehensive planning and virtual surgical simulation.

Advanced AI systems would then be capable of segmenting, measuring, and planning implant dimensions, positions, and angles based on biomechanical and anatomical data, which could improve standard of care. Real-time clinical deployment, interactive interfaces, and explainable AI outputs could facilitate seamless integration into practice, enhancing efficiency and clinician confidence.

## Conclusion

This study demonstrates that a U-Net++–based AI system can accurately segment the alveolar ridge from a CBCT image and produce reliable bucco-lingual bone width measurements. However, mandibular canal segmentation remains a challenge. AI-generated measurements showed moderate agreement with manual assessments, which shows potential to reduce time and improve consistency in implant planning with further training. The results support the system’s overall performance and suggest its feasibility as a clinical decision-support tool. Broader validation across jaw regions, CBCT systems, and patient populations is necessary to ensure generalisability. AI integration in CBCT analysis promises to streamline workflow, enhance measurement accuracy, and support personalised, dental implant planning.

## Data Availability

Data is provided within the manuscript.
